# Non-Leukemic Granulocytic Sarcoma Presenting with Multiple Lymphadenopathies

**DOI:** 10.4274/tjh.2016.0428

**Published:** 2017-06-01

**Authors:** Ayfer Gedük, Esra T. Demirsoy, Süheyla U. Bozkurt, Zafer Gülbaş, Serkan İşgören

**Affiliations:** 1 Kocaeli University Faculty of Medicine, Department of Hematology, Kocaeli, Turkey; 2 Marmara University Faculty of Medicine, Training and Research Hospital, Department of Pathology, İstanbul, Turkey; 3 Anadolu Medical Center, Bone Marrow Transplantation Center, Kocaeli, Turkey; 4 Kocaeli University Faculty of Medicine, Department of Nuclear Medicine, Kocaeli, Turkey

**Keywords:** Granulocytic sarcoma, Leukemia, Lymphadenopathy

## To The Editor,

Granulocytic sarcoma (GS) is a rare tumor with poor prognosis that is composed of primitive myeloid cells, localized in extramedullary sites. The incidence is 2.5%-9.1% in acute myeloid leukemia (AML) patients and it may also occur in association with a myeloproliferative neoplasm or myelodysplastic disorders. The most common locations are the skin, lymph nodes, gastrointestinal tract, bones, and soft tissues [1].

**Figure 1:**
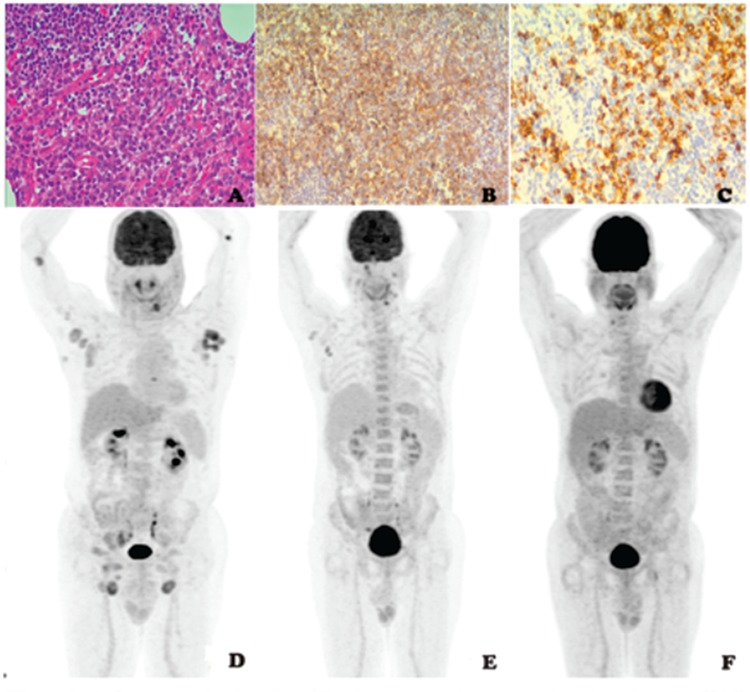
A) Large neoplastic cells infiltrating paracortical areas of the lymph node (hematoxylin and eosin, 400^x^). B) Immunohistochemistry showing CD34 immunoreactivity in the cytoplasm of the neoplastic cells (CD34 stain, 400^x^). C) Immunohistochemistry showing myeloperoxidase immunoreactivity in the cytoplasm of the neoplastic cells (myeloperoxidase stain, 400^x^). D) Initial positron emission tomography/computed tomography (PET/CT) presents multiple hypermetabolic lymph nodes. E) PET/CT after 3+7 regimen shows partial response. F) PET/CT after fludarabine, cytarabine, G-CSF, and idarubicin regimen shows complete metabolic response.

A 68-year-old man presented with the complaint of bilateral inguinal swelling. On physical examination bilateral cervical, axillary, and inguinal multiple lymphadenopathies (LAP), approximately 2-3 cm in diameter, were noted. Initial workup revealed normal liver and renal functions; negative viral serology for epstein-barr virus, cytomegalovirus, HIV, and hepatitis B/C; and mild neutropenia (1020/µL). An excisional LAP biopsy revealed disruption of the normal lymph nodal architecture by a diffuse monomorphic infiltrate comprising medium-sized mononuclear cells with a high nuclear cytoplasmic ratio and fine chromatin pattern. In immunohistochemical study, these cells were positive for CD34, CD43, CD117, and myeloperoxidase and negative for Tdt, CD3, CD5, CD20, CD15, CD30, CD56, EMA, and Pax-5. The Ki-67 proliferation index was 45% and EBER was negative. Leukemic infiltration could not be detected in the bone marrow examination. Cytogenetic analysis was negative for t(9,22), t(15,17), t(8,21), and inv16 but positive for NPM1 and 11q23 rearrangement. The results confirmed the diagnosis of GS (Figures 1A,1B,1C). A positron emission tomography/computed tomography (PET/CT) scan showed multiple hypermetabolic lymph nodes (SUV_max_: 11.8) in bilateral cervical, axillary, paracardiac, and both common iliac areas (Figure 1D). Cytosine-arabinoside plus idarubicin (3+7 regimen) was started. PET/CT was repeated after chemotherapy and revealed partial response (Figure 1E). The persistent disease was confirmed by an excisional LAP biopsy and a fludarabine, cytarabine, G-CSF and idarubicin regimen was started. Since complete metabolic response was detected in the follow-up PET/CT, he underwent a matched related donor reduced-intensity conditioning hematopoietic stem cell transplantation (HSCT) (Figure 1F). He engrafted successfully and has had no recurrent GS for 8 months since the transplant.

Although it is well recognized that GS can cause localized lymphadenopathy, manifestation as bilateral multiple LAP is rare [2]. Nandedkar et al. [3] reported a case of GS presenting with bilateral multiple LAP without evidence of leukemia, which is similar to our patient. In these two cases the interesting point is a myeloid malignancy presenting with only lymphatic spread. There is no consensus on the treatment of non-leukemic GS. Common practice suggests AML-like induction chemotherapies as first-line treatment and allogeneic HSCT for relapsed/refractory disease. Radiotherapy and surgery are also options in selected cases [4]. In a study by Chevallier et al. [5] that assessed the outcome of 30 patients with non-leukemic GS who underwent allogeneic HSCT, 5-year overall survival and leukemia-free survival were 48% and 36%, respectively. In another study, a significantly longer event-free survival rate was detected in patients treated with chemotherapy and allogeneic HSCT [6]. These data suggest that allogeneic HSCT is efficient as a consolidation regimen for GS. In our case, PET/CT was used for disease detection and in the monitoring of treatment response, which is an effective imaging tool for GS [7]. In conclusion, we present a case of GS with lymphatic spread like a lymphoma.
